# Neuropsychological Outcome and Diffusion Tensor Imaging in Complicated versus Uncomplicated Mild Traumatic Brain Injury

**DOI:** 10.1371/journal.pone.0122746

**Published:** 2015-04-27

**Authors:** William J. Panenka, Rael T. Lange, Sylvain Bouix, Jason R. Shewchuk, Manraj K. S. Heran, Jeffrey R. Brubacher, Ryan Eckbo, Martha E. Shenton, Grant L. Iverson

**Affiliations:** 1 Department of Psychiatry, University of British Columbia, Vancouver, Canada; 2 Defense and Veterans Brain Injury Center, Walter Reed National Military Medical Center, Bethesda, Maryland, United States of America; 3 Psychiatry Neuroimaging Laboratory, Brigham Women’s Hospital, Harvard Medical School, Boston, Massachusetts, United States of America; 4 Department of Radiology, University of British Columbia, Vancouver, Canada; 5 Department of Emergency Medicine, University of British Columbia, Vancouver, Canada; 6 VA Boston Healthcare System, Brockton, Massachusetts, United States of America; 7 Department of Physical Medicine and Rehabilitation, Harvard Medical School, Spaulding Rehabilitation Hospital, & Red Sox Foundation and Massachusetts General Hospital Home Base Program, Boston, Massachusetts, United States of America; University of Ulm, GERMANY

## Abstract

This study examined whether intracranial neuroimaging abnormalities in those with mild traumatic brain injury (MTBI) (i.e., “complicated” MTBIs) are associated with worse subacute outcomes as measured by cognitive testing, symptom ratings, and/or diffusion tensor imaging (DTI). We hypothesized that (i) as a group, participants with complicated MTBIs would report greater symptoms and have worse neurocognitive outcomes than those with uncomplicated MTBI, and (ii) as a group, participants with complicated MTBIs would show more Diffusion Tensor Imaging (DTI) abnormalities. Participants were 62 adults with MTBIs (31 complicated and 31 uncomplicated) who completed neurocognitive testing, symptom ratings, and DTI on a 3T MRI scanner approximately 6-8 weeks post injury. There were no statistically significant differences between groups on symptom ratings or on a broad range of neuropsychological tests. When comparing the groups using tract-based spatial statistics for DTI, no significant difference was found for axial diffusivity or mean diffusivity. However, several brain regions demonstrated increased radial diffusivity (purported to measure myelin integrity), and decreased fractional anisotropy in the complicated group compared with the uncomplicated group. Finally, when we extended the DTI analysis, using a multivariate atlas based approach, to 32 orthopedic trauma controls (TC), the findings did not reveal significantly more areas of abnormal DTI signal in the complicated vs. uncomplicated groups, although both MTBI groups had a greater number of areas with increased radial diffusivity compared with the trauma controls. This study illustrates that macrostructural neuroimaging changes following MTBI are associated with measurable changes in DTI signal. Of note, however, the division of MTBI into complicated and uncomplicated subtypes did not predict worse clinical outcome at 6-8 weeks post injury.

## Introduction

A substantial minority of patients who sustain a mild traumatic brain injury (MTBI) show trauma-related intracranial abnormality detected on day-of-injury computed tomography, with prevalence rates varying from 5%[1] to nearly 40%[2] across studies[3]. When examining these studies in more detail the wide range is partially attributable to differential enrollment of patients with lower Glasgow Coma Scale (GCS) scores of 13, and different referral patterns for neuroimaging[3]. In addition to lower GCS scores, other clinical predictors of intracranial Computed tomography [4] abnormalities following adult MTBI include the presence of skull fractures, focal neurological signs, seizures, persistent vomiting, retrograde amnesia, age>60, presence of coagulopathy, high likelihood mechanism (fall from height, pedestrian motor vehicle accident), chronic alcohol use and previous neurosurgical procedure[5]. Magnetic resonance imaging (MRI), if conducted routinely, would yield higher rates of structural abnormalities following MTBI[6,7,8,9]. Those patients whose TBI severity falls in the mild range (e.g., duration of loss of consciousness < 30 min, Glasgow Coma Scale = 13–15, and duration post-traumatic amnesia <24 hours), and who show neuroradiological evidence of a trauma-related intracranial abnormality, have been conceptualized as having a complicated MTBI[10]. Intuitively, we would expect that patients with complicated MTBI would have worse outcome than those with normal imaging following MTBI (i.e., uncomplicated MTBI). However, the literature examining the relation between neuroimaging abnormalities and outcome following MTBI is inconsistent.

A review of studies comparing symptom reporting and functional and cognitive outcomes following MTBI in patients with and without intracranial imaging abnormalities is presented in Table 1. Not included in this list are a number of other studies where complicated or uncomplicated patients are compared solely with control patients,[11,12,13,14] or where imaging abnormalities are examined in the context of broader TBI severity[14,15,16,17].

**Table 1:** Review of studies comparing outcomes between Uncomplicated and Complicated MTBI subjects.

First Author	Year	Country	Adult (A) or Pediatric (P)	Comp MTBI (N)	Uncomp MTBI (N)	Imaging method	MRI magnet strength, timing after injury	Timing of clinical testing	Recruitment location	Neuroimaging Findings Related to Outcome		
										Functional	Cognitive Testing	Symptoms
Williams [10]	1990	USA	A	77	78	CT	NA	NP after clearing of PTA, GOS at 6 months	Neurosurgical service	Complicated less likely to show good recovery on GOS	Complicated worse on tests of processing speed and recognition memory, no difference on verbal fluency or verbal memory	NA
Hanlon [18]	1999	USA	A	22	78	CT, MRI	Not reported	NP mean 177 days post injury, vocational at 1 year.	Outpatient	No difference in vocational outcome	No difference	NA
Iverson [19]	1999	USA	A	112	369	CT	NA	NP—most within 1 week of injury	Trauma Service	NA	Complicated worse on COWAT. No other tests were reported.	NA
Hofman [20]	2001	Netherlands	A	12	9	MRI	1.5T, 2–5 days	NP and Sx evaluation < 1 week, 2 months, and 6 months.	ER	NA	Complicated worse on 1/6 measures—Reaction time at 6 months	No difference
Borgaro [21]	2003	USA	A	14	14	CT, MRI	Magnet strength not reported, < 40 days	NP <40 days	Inpatient Rehabilitation	NA	Complicated worse on 1/7 subsets of the BNIS—speech/language testing	NA
Hughes [22]	2004	UK	A	26	54	MRI	1.0T, 1–3 days	NP < 3 days, vocational status and Sx evaluation at 6 months	ER	No difference on return to work status	Complicated worse on a composite measure of attention, no difference on memory or executive functioning	No difference
Lange [23]	2005	USA	A	117	328	CT	NA	NP < 23 days, most < 7 days	Trauma Service	NA	Complicated worse on TMT-A and TMT-B. Other tests were not reported.	NA
Iverson [24]	2006	USA	A	50	50	CT	NA	NP < 2 weeks	Trauma Service	NA	Complicated worse on a minority of measures (5/13) including TMT-B, COWAT, Logical Memory Saving Score, Visual Reproduction I and II (d<0.51 for all scores)	NA
Kurca [25]	2006	Slovakia	A	7	23	MRI	1.5T, <4 days	NP and Sx evaluation < 4 days	Unclear	NA	Complicated worse on a minority of measures (2/11)—more errors on the CAT and a learning interference test where a list of words is recalled after learning a subsequent list	No difference
Sadowski-Cron [26]	2006	Switzerland	A	21	184	CT	NA	NP < 2 hours, Sx evaluation 1 year	ER	NA	No difference	Complicated reported more symptoms
Lee [9]	2008	USA	A	19	9	CT, MRI	3T, <14 days	NP and GOS-E within 2 weeks, 1 month, and 1 year	ER	No difference on GOS-E	No difference	NA
Levin [27]	2008	Canada/USA	P	32	48	CT	NA	NP at 2 weeks, 3 months, 6 months, and 12 months	Pediatric ER	NA	Complicated worse on 5/7 domains—general working memory, episodic memory, visuomotor speed, reading, and processing speed	NA
Stulemeijer[28]	2008	Netherlands	A	40	133	CT	NA	6 months	ER	No difference RTW	NA	No difference
Lange [29]	2009	USA	A	20	20	CT	NA	NP Average 3.5 days	Trauma Service	NA	Complicated worse on 3/13 measures including Visual Reproduction I and II, and Hopkins Delay (d<.76)	NA
Muller [30]	2009	Norway	A	15	45	CT, MRI	<2 days	NP before discharge and at 6 months	Neurosurgery service	NA	Complicated worse at both time points on “mean impairment index”—a composite measure derived from a comprehensive NP battery	NA
de Guise [31]	2010	Canada	A	45	131	CT	NA	NP at 3 weeks, Sx evaluation at 2 weeks	Outpatient	No difference GOS-E, no difference return to work or discharge referrals pattern	No difference	Uncomplicated reported more symptoms
Iverson [3]	2012	Finland	A	13	34	CT, MRI	3T MRI, 3–4 weeks	NP at 3–4 weeks	ER	Complicated more time to return to work	No difference	No difference
Lange [32]	2012	USA	A	17	24	CT, MRI	Magnet strength not reported, timing in first few weeks	NP within 6 months of injury	Combat evacuated	NA	Uncomplicated group worse on 1/11 measures tested—CVLT-II long delayed recall (d = 0.86)	Uncomplicated worse on a minority of PAI subscales—anxiety (d = .74) and aggression (d<0.72).
Babcock[33]	2013	USA	P	14	206	CT	NA	Symptoms at 3 months	ER	NA	NA	No difference
Beauchamp [34]	2013	Australia	P	13	58	CT, MRI	3T MRI, 2–8 weeks	NP, outcome measures at 6 months	ER	Global adaptive functioning—no difference	No difference	NA
Dagher [35]	2013	Canada	A	1,483	644	CT	NA	Outcome measures at discharge	Inpatient	Complicated longer length of stay, worse on GOS-E and FIM	NA	NA
McMahon [36]	2013	USA	A	165	210	CT, MRI	NA	Sx and outcome evaluation at 3,6, and 12 months	ER	Complicated worse on GOS-E at 3 months, no difference at 6 or 12 months	NA	Uncomplicated reported more symptoms
Lannsjo[37]	2013	Sweden	A	52	1210	CT	NA	3 months	ER	No difference on GOSE	NA	No difference
Kumar[38]	2014	USA	A	71	57	CT	NA	3 months	ED	NA	NA	No difference
Yuh [8]	2013	USA	A	64	71	CT, MRI	3T, average 12 days	GOS-E at 3 months	ER	Group with any CT abnormality showed no difference on GOS-E. Note: Subgroup of patients with Diffuse axonal injury, contusions, or subarachnoid hemorrhage worse on GOS-E at 3 months)	NA	NA
Yuh[39]	2014	USA	A	32	44	CT, MRI	3T	3 and 6 months	ED	No difference GOS-E Note: Subgroup with MRI contusions associated with worse outcome at 3 months only (see text).	No difference on CVLT-II, WAIS-IV-PSI, TMT-A, TMT-B	No difference

Abbreviations: Comp = Complicated; Uncomp = Uncomplicated; NP = Neuropsychological testing; PTA = Post-Traumatic Amnesia; Sx = symptoms, ER = emergency room, GOS = Glasgow Outcome Scale; GOS-E = Glasgow Outcome Scale Extended, FIM = Functional Independence Measure; RT = Reaction Time; COWAT = Controlled Oral Word Association Test; TMT = Trail Making Test; BNIS = Neuropsychological Instrument BNI Screen for Higher Cerebral Functions; CVLT-II = California Verbal Learning test II; CAT = Concentration and Attention Test; SF-36 = Short-Form health survey; HI-FI = Head Injury Family Interview Checklist; WAIS IV-PSI = Weschler Adult Intelligence Scale IV edition processing speed index, RPQ = Rivermead Post-Concussion Symptom Questionnaire

A careful review of the literature lends some support to a negative relationship between the presence of intracranial abnormality and neurocognitive test performance in MTBI subjects (Table 1, 11 of 19 studies—58%); but in several of these studies the relationship is modest. In most studies that do show a relationship the effect sizes are smaller than expected, and significance is obtained in only a subset of the cognitive battery employed[32].

Some studies suggest that complicated MTBI patients have different functional outcomes than uncomplicated patients. In 4 of 13 studies that reported on outcome (see Table 1), complicated MTBI patients had greater problems as measured by the Glasgow Outcome Scale, the Functional Independence Measure, the Global Adaptive Functioning Scale, or return to work. Most studies, however, have not found a significant difference in functional outcomes (see Table 1).

This is also the case for symptoms. Paradoxically there are more studies in the literature suggesting that complicated MTBI patients *report fewer symptoms* as there are studies suggesting that complicated MTBI patients *report more symptoms*. The most consistent symptomatic finding in the complicated/uncomplicated literature is that these two groups are indistinguishable in terms of symptom reporting, with 9 of the 13 studies not showing a significant difference (see Table 1).

Diffusion tensor imaging (DTI) is now commonly used in MTBI research,[6,40] but it has not been used explicitly to compare those with complicated versus uncomplicated MTBI. In DTI, the white matter tracts are analyzed in-vivo by inference from the local diffusion of water molecules. Commonly measured DTI parameters include fractional anisotropy (FA), mean diffusivity (MD), radial diffusivity (RD), and axial diffusivity (AD)[40].FA is a representation of the directional coherence (anisotropy) of water diffusion in the tissue, and is thought to be proportional to the anatomical integrity of white matter tracts[40]. MD represents the average total diffusion of water within a particular voxel, irrespective of direction, and in general MD values are found to be increased in response to white matter injury.[40] RD represents the diffusion of water perpendicular to the axon, and thus is thought to be sensitive to processes such as demyelination.[41] AD represents the diffusivity parallel to the axon and hence changes in AD are thought to indicate axonal compromise[41].

The anatomical areas showing DTI abnormalities in those patients who have sustained MTBI vary considerably among studies. The inherently heterogeneous nature of brain trauma, methodological differences such as time of scanning after injury, magnet strength, and differences in post-processing techniques all account for part of this variance. Some anatomical areas, however, appear to be consistently reported as more vulnerable to the effects of MTBI. According to a meta-analysis of DTI findings in MTBI, the corpus callosum (CC) is the structure[42] most consistently reported as abnormal. A recent systematic review of DTI findings in sport concussion concluded that the CC, internal capsule, and the longitudinal fasciculi were the most frequently reported areas with abnormalities[43]. The frontal association pathways, including the uncinate fasciculus, superior longitudinal fasciculus, and anterior corona radiata may also be vulnerable[44]. The predominant subacute-chronic DTI finding is a decrease in FA, an increase in MD, and radial and axial values that following a more complicated temporal course[41]. Several DTI studies show a correlation between DTI metrics and neurocognitive testing[40]. The data on post-concussive symptoms and DTI measurements, however, is mixed, with some studies showing a correlation,[45,46,47,48,49] and other studies not showing a significant association[50,51]. For a comprehensive review of DTI findings in MTBI, and their relation to neurocognition and symptoms, the reader is referred to three recent works[6,12,40].

The purpose of this study is to examine the influence of trauma-related intracranial abnormalities, as evidenced by CT and MRI, on subacute neurocognitive outcome, post-concussion symptom reporting, mental health, and the microstructural architecture of white matter. We hypothesized that (i) the complicated MTBI group would report more symptoms and have worse neurocognitive outcomes than the uncomplicated MTBI group, and (ii) the complicated MTBI group would show a greater degree of white matter compromise, most notably in the region of the CC, compared to the uncomplicated MTBI group.

## Materials and Methods

### Research Ethics

This study was approved by the Clinical Research Ethics Board at the University of British Columbia, Vancouver, Canada. All participants gave written informed consent prior to participation.

### Participants

Participants were 62 patients with MTBI (31 uncomplicated and 31 complicated) who were recruited (between 2007 and 2012) from the Emergency Department of Vancouver General Hospital (VGH), British Columbia, Canada. Participants were included in the MTBI group if they (a) presented to the Emergency Department following head trauma, and (b) had evidence of a closed TBI as indicated by at least one of the following: (a) witnessed loss of consciousness (LOC) of at least one minute duration, (b) post-traumatic amnesia (PTA) of more than 15 minutes, or (c) Glasgow Coma Scale (GCS) score = 13–14. Classification of brain injury severity was based on the duration of loss of consciousness (LOC), duration of post-traumatic amnesia (PTA), GCS scores, day-of-injury CT scans, and structural MRI scans 6–8 weeks post-injury as follows: (a) uncomplicated mild TBI: LOC <30 minutes, GCS = 13–15, PTA <24 hours, and no trauma-related intracranial abnormality on day-of-injury CT or 6–8 week structural MRI scan; (b) complicated mild TBI: LOC <30 minutes, GCS = 13–15, PTA <24 hours, and trauma-related intracranial abnormality on day-of-injury CT scan and/or 6–8 weeks later on structural MRI scan. Of the 31 patients classified as complicated injuries, 16 had abnormalities on day-of-injury CT and 15 did not (the 15 were identified on MRI at 6–8 weeks following injury). MTBI criteria as set forth by the World Health Organization working group[52] and the American Congress of Rehabilitation Medicine Special Interest Group[53] were met for all subjects, but the criteria for MTBI in this study were more rigorous (see above) than both of those diagnostic systems to avoid enrolling false positives or those with equivocal mild injuries.

A control sample of 32 patients was recruited from the Emergency Department of VGH and they were initially included if (1) they sustained a soft-tissue or orthopedic injury below the neck; (2) there was no evidence of an altered state of consciousness as indicated by a reduction in GCS score, or presence of a LOC, PTA, or post-traumatic confusion; and (3) there was no evidence of physical head trauma, whiplash, or cervical strain based on medical chart review (e.g., absence of lacerations/contusions to the head, absence of complaints of head, neck, or back pain).

These patients represent a subset from a larger longitudinal cohort MTBI study that began recruiting in June of 2007. VGH emergency department records were screened for trauma admissions between the ages of 19 and 55, who also had a blood alcohol level (BAL) measured on the day of injury. Exclusion criteria included the inability to communicate effectively in English; educated in a language other than English after age 10; history of serious neurological disorder, TBI, learning disability, or psychiatric illness resulting in hospitalization; presence of MRI contraindications; significant drug abuse other than alcohol; restricted use of hands/arms for neuropsychological testing; or serious difficulties with eyesight.

Following medical chart review, patients without obvious exclusion criteria were contacted and assessed for their willingness to participate and to undergo a comprehensive review of inclusion/exclusion criteria. English language difficulties, mental or neurological disorder, illicit drug use, equivocal TBI (for TBI group only), contraindication for MRI, and presence of learning disability or attention-deficit hyperactivity disorder were frequent reasons for exclusion. Participants in the TBI group that agreed to participate did not differ from those who declined in age, day of injury BAL, GCS scores (at site of injury or on admission to hospital), gender, or presence of intracranial abnormality. Complicated and uncomplicated MTBI groups were created by querying the full database for subjects with MTBI.

#### Participant Selection

Participants were selected from a sample of 170 patients enrolled in a larger study (105 MTBI and 65 TC) in a three-step manner. First, participants were initially included in the sample if they met the following criteria: (1) completed the entire neuropsychological test battery; (2) neurocognitive test performance was not considered influenced by reduced English language proficiency; (3) scored above the recommended cutoff for good effort on the Test of Memory Malingering; (4) behavioral observations during the neuropsychological evaluation did not provide suspicion of questionable motivation or inattention that may have negatively influenced test performance; (5) successfully completed MRI scanning; (6) there was no evidence of obvious incidental neurological abnormalities on MRI such as meningioma, cistern mass, venous anomaly; and (7) structural MRI scans were considered complete and readable by a neuroradiologist. In addition, in the MTBI group, participants were only included if (8) there was no evidence of intracranial abnormality that was considered to pre-date the current injury. Similarly, in the TC group, participants were only included if (9) they had maximum of one white matter hyperintensity visible on MRI (WMHI) because it has been reported that having two or more white matter hyperintensities is associated with DTI abnormalities in brain regions distal from these abnormalities[54,55]. The application of these criteria resulted in retaining 127 participants [40 TC and 87 MTBI (i.e., 41 uncomplicated MTBI, 46 complicated MTBI)].

Second, in order to control for the influence of age and pre-injury alcohol consumption, participants were further excluded if they were 50 years or older or if their pre-injury alcohol consumption was considered to be unusually high. Unusually high pre-injury alcohol consumption was defined as drinking more than 30 drinks per week and/or binge drinking more than four or more days per week (binge drinking = 5 or more drinks on any one occasion). This level of alcohol consumption, which is liberal, was chosen in part because the dementia literature occasionally defines up to four drinks/day as moderate, and “very heavy drinking” as at least five drinks/day (for a recent meta-analytic review see Anstey et al, 2009[56]). The application of these criteria resulted in retaining 110 participants [36 TC and 74 MTBI (i.e., 38 uncomplicated MTBI, 36 complicated MTBI)].

Third, prior to DTI post-processing, the MRI scans were inspected for image quality. Scans were dropped if they exhibited significant motion artifact or brain coverage was inadequate. The application of these criteria resulted in retaining a final sample of 94 participants [32 TC subjects and 62 MTBI subjects (i.e., 31 uncomplicated MTBI, 31 complicated MTBI)].

### Measures

Participants completed a 5-hour neuropsychological assessment battery that included measures of neurocognitive functioning, post-concussion symptoms, mental health, and substance use history approximately 6–8 weeks post injury (M = 47.1 days, SD = 5.6). All participants gave written informed consent in accordance with the Clinical Research Ethics Board at the University of British Columbia, Vancouver, Canada.

#### Symptom Measures

Participants completed the Beck Depression Inventory-Second Edition (BDI-II), Beck Anxiety Inventory [57], and the British Columbia Post-concussion Symptom Inventory (BC-PSI). The BDI-II[58] and BAI[59] are both 21-item self-report questionnaires designed to assess depressive and anxiety symptoms respectively. Participants are asked to rate each item on a four-point scale ranging from 0 to 3. A total score is calculated by summing all 21 items on each measure separately, giving a total score on each measure with a range from 0 to 63.

The British Columbia Post-Concussion Symptom Inventory (BC-PSI)[60,61] is based on ICD-10 criteria[62] for Postconcussional Syndrome and requires the test taker to rate the frequency and intensity of 13 symptoms (i.e., headaches, dizziness/light-headedness, nausea or feeling sick, fatigue, sensitivity to noises, irritability, sadness, nervousness/tension, temper problems, poor concentration, memory problems, reading difficulty, and sleep disturbance) as well as the effect of three co-occurring life problems on daily living (i.e., greater present versus past effects of alcohol consumption, worrying and dwelling on symptoms, and self-perception of brain damage). The three life problems are rated on a scale from one to five, where 1 = “not at all” and 5 = “very much”. The 13 symptoms are rated on a six-point Likert-type rating scale that measures the frequency (i.e., “how often”) and intensity (“how bad”) of each symptom in the past two weeks. Frequency ratings range from 0 = “not at all” to 5 = “constantly”. Intensity ratings range from 0 = “not at all” to 5 = “very severe problem”. For each of the 13 symptoms, the two ratings are multiplied together (how often x how bad) to create a single score for each item. These product-based scores are then converted to item scores that reflect both the frequency and intensity of symptom endorsement (range = 0 to 4). Item scores of 1 are interpreted as falling in the mild range. Item scores of 3 are interpreted as falling in the moderate range.

#### Neurocognitive Measures

The neurocognitive measures consisted of 16 tests from the Neuropsychological Assessment Battery (NAB;[63]). The NAB is a comprehensive, co-normed (across all tests) neuropsychological test battery that consists of 24 individual tests designed to assess cognitive functioning across five domains: Attention, Language, Memory, Spatial, and Executive Functioning. The normative sample is large and the coverage of neuropsychological abilities assessed is broad. The NAB can be used in a fixed or flexible manner. Only 16 of the 24 tests were selected for use in order to reduce administration time. The administration of the 16 selected tests results in the acquisition of 23 scores of interest. In order to reduce the number of cognitive variables for the analyses, the 23 scores of interest were used to generate index scores for each of the five cognitive domains. The Attention and Memory indexes were generated as per the instructions in the manual. For the Language, Spatial, and Executive Functioning Indexes, however, not all subtests included in these indexes were administered. As such, these indexes were prorated. These three indexes were calculated by generating a prorated ‘Sum of T-scores’ and then converting the prorated ‘Sum of T-scores’ to a standard score using the look-up table (i.e., Table 7.1) in the NAB normative manual[64]. The prorated Sum of T-scores for the three indexes was calculated by averaging the demographically adjusted T-scores across all available subtests that are included in each index. The mean T-score was then multiplied by the number of subtests that are used to generate the full version of the index. For the Language Index, two of five possible subtests were used (i.e., Oral Production and Naming). For the Spatial Index, two of four possible subtests were used (i.e., Visual Discrimination and Design Construction). For the Executive Functioning Index, three of four possible subtests were used (Categories, Mazes, and Word Generation). A NAB Total Index was also generated using the standard scores derived for the five indexes above as per the instructions in the manual.

Participants were also administered the Test of Memory Malingering[65] to evaluate the possibility of the patient providing poor effort during testing. No subject was excluded due to poor effort.

### Neuroimaging

MRI data were acquired on a Philips Achieva 3T scanner with Dual Nova Gradients (maximum gradient strength 80 mT/m, maximum slew rate 200 mT/m/s) and an 8-channel phased array head coil in parallel imaging mode. Diffusion tensor imaging data were acquired using an eddy current compensated, single-shot, spin-echo, echo planar imaging sequence with unipolar diffusion weighting along 16 noncollinear directions and a maximum b value of 1000 s/mm2. Further DTI parameters were as follows: acquisition matrix 96*96, 50 contiguous slices, 2.5*2.5*2.5mm isotropic acquisition resolution, time to echo 75 ms, time to repetition 5600 ms, parallel imaging SENSE-factor = 2.4.

The structural T1 and FLAIR images were reviewed by a board certified radiologist (JRS or MKSH) who determined whether any visible trauma related intracranial pathology was present. Pertinent MRI findings that were coded for all patients included the number and location of susceptibility foci, T2 hyperintensities, encephalomalacia, and extra-axial collections

#### Tract-based Spatial Statistics

(TBSS[66] and Randomise[67] part of the FMRIB Software Library (FSL, Analysis Group, FMRIB, Oxford, UK)[68] was used for comparisons of FA, MD, AD and RD. DTI sequences with poor field-of-view resulting in significant deficits in whole brain coverage or artifacts were not considered in the analysis. Motion and artifacts in the diffusion data were corrected using affine registration of all gradient volumes with the b = 0 volume (FLIRT; FMRIB Software Library, Oxford, UK), and gradient directions were compensated for rotations[69]. This was followed by creation of a manual brain mask based on the b = 0 image using 3D Slicer version 2.7[70]. Individual FA maps were then non-linearly registered via FSL-FNIRT to the JHU-ICBM FA template provided by FSL, followed by creation of a mean FA skeleton. Individual FA values were then projected onto this mean skeleton. MD, AD, and RD values were projected onto the skeleton using the previously created FA transformation. FA threshold was set to 0.25 and voxel-based comparisons of FA, MD, AD, and RD were performed on the TBSS skeleton using Randomise [67]from FSL Version 4.1.9[66]. with the number of permutations was set at 5000 and threshold-free cluster enhancement (TFCE) with correction for family wise error rate employed. The p<.05 T-contrast maps were projected on the mean FA images. To identify which specific anatomical areas were implicated, the mean FA skeleton and contrast map were overlaid with the John Hopkins University (JHU) International Consortium Brain Mapping (JHU-ICBM-DTI-81) white matter label atlas[71,72,73].

FSL statistics were then used to compute the mean FA values for each individual for each of the regions of interest (ROIs) on the mean FA skeleton. Mean values for MD, AD, and RD were done similarly. Forty-eight individual ROIs were identified according to the International Consortium of Brain Mapping (ICBM) DTI-81 white-matter labels atlas[71,72,73].

The ROIs from the ICBM-DTI-81 atlas included the (a) genu, body, and splenium of corpus callosum; (b) pontine crossing tract, fornix, and middle cerebellar peduncle; and (c) symmetrical ROIs (left/right) each for the corticospinal tract, medial lemniscus, inferior cerebellar peduncle, superior cerebellar peduncle, cerebral peduncle, anterior limb of internal capsule, posterior limb of internal capsule, retrolenticular part of internal capsule, anterior corona radiata, superior corona radiata, posterior corona radiata, posterior thalamic radiation, sagittal stratum, external capsule, cingulum (cingulate gyrus), cingulum (hippocampus), fornix/stria terminalis, superior longitudinal fasciculus, superior fronto-occipital fasciculus, uncinate fasciculus, and tapetum.

Due to the large number of ROIs, four summary scores were calculated for each participant and used in all statistical analyses. The four summary scores represent the number of ROIs with FA, MD, AD, and RD values that fell below/above a specified cutoff score for each participant. Cutoff scores were identified by calculating the means and standard deviations (SD) for FA, MD, AD, and RD values in each of the 48 ROIs based on DTI of 36 participants who were recruited for this study, that had sustained an orthopedic injury, but not a TBI. FA values that were >2 SDs below the mean, and MD, AD, and RD values that were >2 SDs above the mean, were classified as reflecting an ROI with “compromised white matter” (i.e., abnormal score).

## Results

### Comparisons between Uncomplicated and Complicated MTBI Groups

Descriptive statistics and group comparisons (using ANOVA and Chi square analyses) of demographics and injury characteristics are presented in Tables 2 and 3. The groups did not differ significantly in age, education, gender, ethnicity, mechanism of injury, days tested post injury, lowest GCS score, duration of LOC, pre-injury alcohol intake, current intellectual ability, or estimated pre-morbid intellectual ability. There was, however, a significant difference for duration of PTA (p = .026). Participants in the complicated MTBI group were more likely to have a longer duration of PTA (96.8%) compared to the uncomplicated MTBI group (74.2%). Although not significantly different (p = .052), there was a medium effect size for day-of-injury BAL (d = .51, medium effect size), with the uncomplicated group having a slightly higher BAL on presentation to the Emergency Department.

**Table 2:** Descriptive statistics, group comparisons, and effect sizes of demographic and injury severity characteristics (continuous variables) by group.

	Uncomplicated MTBI		Complicated MTBI				
	M	SD	M	SD	p	D	Effect Size
Age (in years)	30.2	7.9	31.0	9.5	.696	.10	Small
Education (in years)	14.8	2.3	14.9	2.2	.825	.06	Very small
Day-of-injury BAL	28.5	29.1	15.0	24.2	.052	.51	Medium
Days tested post injury	46.0	5.5	48.1	5.6	.142	.38	Small-medium
Lowest GCS >30 minutes	14.4	0.8	14.3	0.7	.721	.09	Very small
RIST Index	109.7	10.6	108.3	7.2	.521	.17	Small
WTAR Score	107.5	7.8	106.7	7.0	.669	.11	Small
Premorbid IQ (D+WTAR)	111.0	8.2	110.5	7.4	.795	.07	Very small

Note: N = 62 (Uncomplicated MTBI, n = 31; Complicated MTBI, n = 31); Abbreviations: BAL = blood alcohol level; GCS = Glasgow Coma Scale; RIST = Reynolds Intellectual Screening Test; IQ = Intelligence Quotient; WTAR = Wechsler Test of Adult Reading, D+WTAR is demographic information combined with the reading score to estimate Full Scale IQ.

**Table 3:** Descriptive statistics and group comparisons of demographic and injury severity characteristics (categorical variables) by group.

	Uncomplicated MTBI		Complicated MTBI		
	N	%	n	%	*X* ^2^
Gender					
Male	23	74.2	23	74.2	1.00
Female	8	25.8	8	25.8	
Ethnicity					
Caucasian	26	83.9	26	83.9	1.00
Asian/East-Indian/Other	5	16.1	5	16.1	
Mechanism of Injury					
MVA	11	35.5	13	41.9	.602
Fall	7	22.6	8	25.8	.767
Sports	1	3.2	2	6.5	.554
Assault/Head Blow	6	19.4	2	6.5	.129
Bicycle	6	19.4	6	19.4	1.00
Pre-Injury Alcohol_1_					
Low-Moderate	13	41.9	11	35.5	.602
Heavy	18	58.1	20	64.5	
Day-of-Injury BAL					
Sober (<21 mmol/L)	14	45.2	21	67.7	.073
Intoxicated (≥21 mmol/L)	17	54.8	10	32.3	
LOC					
None	3	9.6	2	6.5	.355
Transient	6	19.4	6	19.4	
1 to 30 minutes	22	71.0	23	74.2	
PTA					
Less than 15 minutes	8	25.8	1	3.2	.026
Greater than 15 minutes	23	74.2	30	96.8	
GCS					
15	17	54.8	13	41.9	.309
13–14	14	45.2	1	58.1	

Note: N = 62 (Uncomplicated MTBI, n = 31; Complicated MTBI); Abbreviations: CT = computed tomography; GCS = Glasgow Coma Scale; PTA = post-traumatic amnesia; LOC = loss of consciousness; MTBI = mild traumatic brain injury; MVA = motor vehicle accident; BAL = blood alcohol level. Footnotes: _1_ Defined based on criteria for heavy drinking established by the National Institute on Alcohol Abuse and Alcoholism: (a) Females: 8 or more drinks per week or 4 or more drinks on a single occasion more than 52 times per year; (b) Males: 15 or more drinks per week or 5 or more drinks on a single occasion more than 52 times per year.

Of the thirty one participants in the complicated MTBI group, fifteen participants had evidence of trauma-related intracranial abnormalities on day-of-injury CT scan. Of these fifteen participants eleven also had trauma-related intracranial abnormalities on follow-up MRI. The other sixteen participants in the complicated group had trauma-related abnormalities on follow-up MRI, but not on their day-of-injury CT scan. The traumatic abnormalities identified on MRI were as follows: greater than five susceptibility foci in sixteen subjects, two to five susceptibility foci in severn subjects, and one susceptibility focus in four subjects. Traumatic abnormalities identified on CT included parenchymal contusions in six subjects, subarachnoid hemorrhage in nine subjects, subdural hemorrhage in four subjects, and diffuse axonal injury in two subjects.

By group definition, no patient in the uncomplicated group had abnormalities that could be attributed to the trauma on either day-of-injury CT scan (ordered for 30 of the 31 uncomplicated MTBIs), or subsequent MRI. Subgroup analyses between those with CT and MRI abnormalities, versus those with only MRI abnormalities, were not undertaken, however, due to the small sample sizes.

### Neurocognition and Symptom Reporting

Descriptive statistics, group comparisons, and effect sizes for three self-report measures and the six NAB indexes are presented in Table 4. There were no significant differences (using Mann-Whitney U-tests due to a non-normal distribution) between groups on any of the self-report measures including post-concussion symptom reporting (p = .307), anxiety symptoms as measured by the Beck Anxiety Inventory (p = .521), or depressive symptoms as measured by the Beck Depression Inventory-Second Edition (p = .450).

**Table 4:** Descriptive statistics, group comparisons, and effect sizes for self-report measures and NAB indexes.

	Uncomplicated MTBI		Complicated MTBI				
	M	SD	M	SD	p	d	Effect Size
*Self-Report Measures*							
Postconcussion (BC-PSI)	10.4	10.1	12.8	10.7	.307	.23	Small
Anxiety [57]	8.5	9.7	8.8	7.3	.521	.03	Very small
Depression (BDI-II)	9.7	10.7	10.8	9.2	.450	.10	Very small
*Neurocognitive Indexes*							
NAB Total Index	107.0	14.7	105.9	12.5	.753	.08	Very Small
NAB Attention Index	106.3	10.1	105.4	15.3	.792	.07	Very Small
NAB Memory Index	100.4	14.0	103.2	12.7	.412	.21	Small
NAB Language Index*	105.1	19.0	103.4	18.3	.724	.09	Very Small
NAB Spatial Index*	108.1	18.0	107.2	14.4	.828	.06	Very Small
NAB Executive Index*	106.9	16.6	104.4	14.7	.535	.16	Small

Note: N = 62 (Uncomplicated MTBI, n = 31; Complicated MTBI, n = 31);

*Cohen’s [74] effect size (d): small (.20), medium (.50), large (.80).

*Prorated Index scores.

Similarly, there were no significant differences (using ANOVA) between groups on the NAB Total Index (p = .753, d = .08, very small effect size) or any of the five NAB Index or prorated Index scores (range: p = .535 to p = .828; d = .06 to d = .21; very small to small effect sizes). Table 5 presents the results of exploratory analyses comparing groups on all of the primary individual test scores from the NAB revealed no statistically significant differences between groups on any of the 23 measures (all p>.05). Very small (e.g., d<0.15) to small (e.g., 0.15<d<0.35) effect sizes were found on the majority of measures, with the exception of three measures that had medium effect sizes (e.g., d>0.35). The complicated MTBI group had non-significantly better scores on Digits Forwards (d = .45) and Daily Living Memory (d = .43) compared to the uncomplicated MTBI group. In contrast, the uncomplicated MTBI group had non-significantly better scores on Word Generation (d = .35) compared to the complicated MTBI group (p = .083 to p = .181).

**Table 5:** Descriptive statistics, group comparisons, and effect sizes for individual NAB tests (demographically-adjusted T scores).

	Uncomplicated MTBI		Complicated MTBI				
	M	SD	M	SD	p	d	Effect Size
Oral Production	50.6	8.5	50.5	8.6	.953	.02	Very Small
Naming	52.5	8.4	51.0	9.8	.532	.16	Small
Visual Discrimination	52.0	8.6	51.7	7.5	.888	.04	Very Small
Design Construction	54.5	9.6	54.6	9.1	.935	.02	Very Small
Digits Forwards	50.0	7.2	53.7	9.0	.083	.45	Medium
Digits Backwards	53.0	6.0	54.2	9.6	.538	.16	Small
Dots	57.6	7.1	56.3	7.7	.494	.18	Small
Driving Scenes	51.3	10.7	49.9	8.9	.589	.14	Very Small
N&L Efficiency Part D	51.4	10.1	50.4	13.3	.740	.09	Very Small
N&L Efficiency Part A	54.1	9.3	52.9	10.5	.647	.12	Very Small
N&L Efficiency Part B	54.4	7.1	53.0	9.4	.524	.16	Small
N&L Efficiency Part C	50.1	8.2	48.2	9.2	.410	.21	Small
List Learning Immediate	49.9	8.6	50.8	8.6	.691	.10	Very Small
Shape Learning Immediate	53.1	9.6	54.5	7.4	.534	.16	Small
Daily Living Immediate	47.8	9.4	51.9	9.6	.095	.43	Medium
Story Learning Immediate	49.7	9.1	50.9	9.9	.622	.13	Very Small
List Learning Delay	52.2	12.0	53.5	10.9	.652	.12	Very Small
Shape Learning Delay	52.4	8.8	53.0	9.4	.792	.07	Very Small
Daily Living Delay	46.1	13.0	48.3	9.5	.452	.19	Small
Story Learning Delay	50.5	6.3	50.6	7.4	.956	.01	Very Small
Mazes	53.0	7.3	52.0	7.4	.594	.14	Small
Categories	52.9	10.2	54.0	9.0	.673	.11	Very Small
Word Generation	53.5	11.2	50.0	8.7	.181	.35	Medium

Note: N = 62 (Uncomplicated MTBI, n = 31; Complicated MTBI, n = 31)

*Cohen’s [74] effect size (d): small (.20), medium (.50), large (.80).

The neurocognitive measures were further examined by calculating the number of low scores across the entire battery of tests. Low scores were defined as demographically-adjusted T-scores less than 40 (below the 16^th^ percentile) or less than 37 (below the 10^th^ percentile). The frequency distributions of individuals, based on number of low scores are presented in Table 6. For example, using the 16^th^ percentile cutoff, 9.7% of subjects (3 individuals) in the complicated group had low scores on six or more of the neurocognitive variables listed in Table 5, while this was true of 3.2% (one individual) of the subjects in the uncomplicated group. As another example, using <10^th^ percentile as the cutoff, 25.8% of subjects in the complicated MTBI group, compared to 12.9% of those in the uncomplicated MTBI group, had low scores on three or more of the neurocognitive variables. This trend toward a greater number of low scores in the complicated MTBI group was not statistically significant using chi-square analysis. No difference in any of these values, at either percentile cutoff or at any number of base rate of low scores, was significant at the p<.05 level.

**Table 6:** Base rate of low scores by group: Individual NAB tests.

	<16^th^ percentile			<10^th^ percentile		
*# Abnormal DTI scores*	*Uncomplicated MTBI*	*Complicated MTBI*	*% Diff*	*Uncomplicated MTBI*	*Complicated MTBI*	*% Diff*
8 or more	—	3.2	3.2	—	—	—
7 or more	3.2	6.5	3.3	3.2	—	3.2
6 or more	3.2	9.7	6.5	3.2	—	3.2
5 or more	9.7	12.9	3.2	6.5	—	6.5
4 or more	22.6	22.6	0	9.7	6.5	3.2
3 or more	25.8	35.5	9.7	12.9	25.8	12.9
2 or more	45.2	54.8	9.6	32.3	41.9	9.6
1 or more	80.6	77.4	3.2	67.7	61.3	6.4
0 scores	100.0	100.0	—	100.0	100.0	—

### Diffusion Tensor Imaging

**Fig 1:**
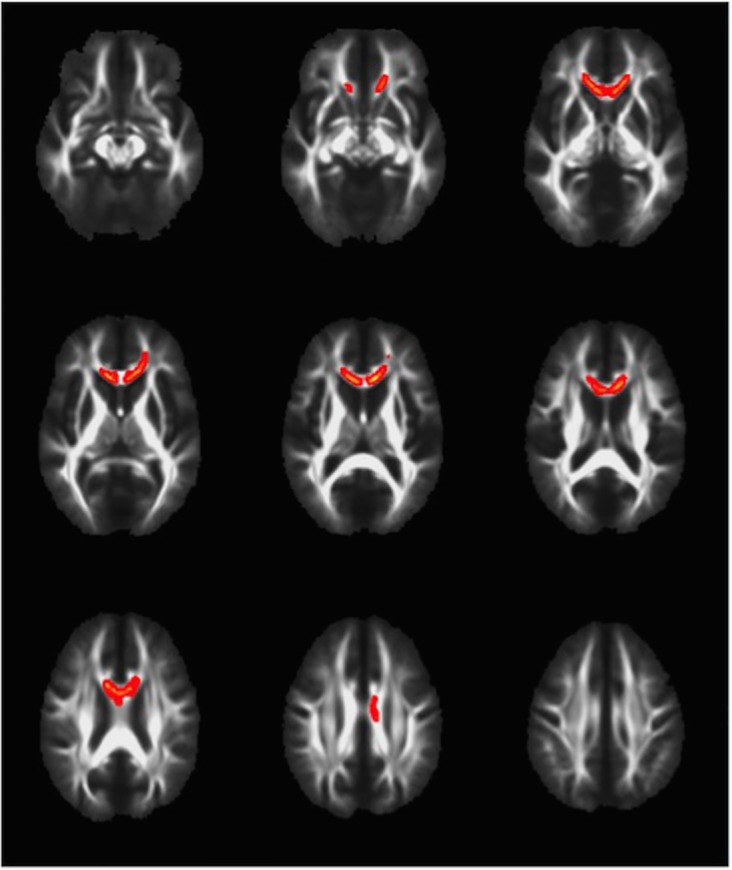
TBSS demonstrates that Fractional Anisotropy is decreased in complicated vs. uncomplicated MTBI. Voxels highlighted in yellow/orange indicate areas of decreased fractional anisotropy (p<0.05) in the complicated MTBI compared to the uncomplicated MTBI group. Neuroanatomical areas where significant differences were found include the body and genu of the Corpus Callosum and left frontal Corona Radiata.

**Fig 2:**
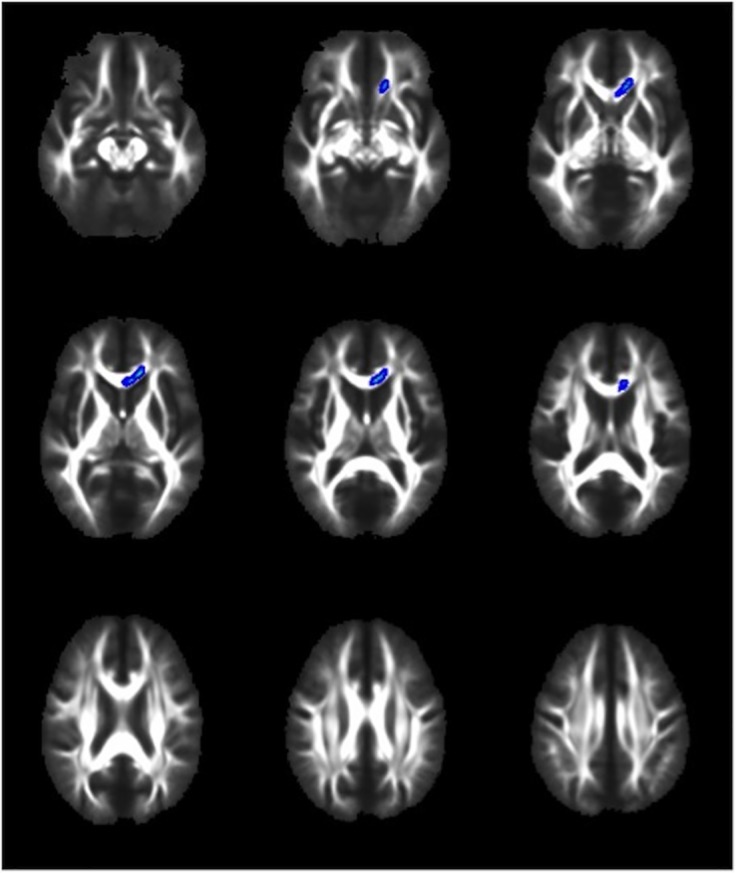
TBSS demonstrates that radial diffusivity is increased in complicated vs. Uncomplicated MTBI. Voxels highlighted in blue/light blue indicate areas of increased radial diffusivity (p<0.05) in the complicated MTBI compared to the uncomplicated MTBI group. Neuroanatomical areas where significant differences were found include the genu of the Corpus Callosum and a small area of the left frontal Corona Radiata.

When comparing the complicated and uncomplicated groups, no significant difference was found for the TBSS measures of MD or AD (data not shown). TBSS analysis did, however, highlight several brain regions that were different on the measures of FA and RD (Figs 1 and 2). FA was significantly decreased in the genu and body of corpus callosum and left frontal corona radiata in the complicated group as compared to the uncomplicated group at the conventional p<0.05 level (Fig 1). In addition, the complicated group showed a significantly increased RD signal in the genu of the corpus callosum and a small area of the left frontal corona radiata at the p<0.05 level (Fig 2). If a more conservative p value (.01) is used given the four analyses, than no significant differences were identified between groups for the TBSS parameters.

Based on ROI analyses, the FA scores for the two groups were compared for the genu (p = .007, d = .71) and body (p = .339, d = .25) of the corpus callosum, and the left anterior (p = .018, d = .62), superior (p = .289, d = .27), and posterior (p = .273, d = .28) corona radiata. In addition, RD scores for the two groups were compared for the genu (p = .009, d = .70) and the left anterior (p = .038, d = .54), superior (p = .225, d = .31) and posterior (p = .489, d = .18) corona radiata. For all ROIs, lower FA values and higher RD values were found in the complicated MTBI group compared to the uncomplicated MTBI group—although some of the ROI findings were not significantly different.

A multivariate region of interest analysis, based on 48 regions, was conducted by determining the number of abnormal areas of white matter as defined by scores that were greater than two standard deviations from the means of the trauma control group. Descriptive statistics, group comparisons, and effect sizes[74] for the number of abnormal areas of FA, MD, AD, and RD by group, are presented in Table 7. There were no significant main effects (using Mann Whitney U tests) for the number of abnormal FA, MD, AD, and RD scores across the two groups. Very small effect sizes were found for all comparisons, with the exception of a small effect size noted for MD.

**Table 7:** Descriptive statistics, group comparisons, and effect sizes for the 48 ROI DTI scores.

	Uncomplicated MTBI				Complicated MTBI						
	M	SD	Median	IQR	M	SD	Median	IQR	p	d	Effect Size
Fractional Anisotropy	1.9	3.3	1	0–2	1.7	2.9	1	0–2	.881	.06	Very Small
Axial Diffusivity	1.3	2.1	0	0–2	1.3	1.6	1	0–2	.668	.02	Very Small
Radial Diffusivity	2.4	4.8	1	1–2	2.5	4.7	1	0–3	.912	.03	Very Small
Mean Diffusivity	1.5	3.0	1	1–2	2.3	4.3	0	0–4	.951	.22	Small

Note: N = 62 (Uncomplicated MTBI, n = 31; Complicated MTBI, n = 31);

*Cohen’s [74] effect size (d): small (.20), medium (.50), large (.80). IQR = Interquartile Range. M = Mean number of ROIs per subject (out of 48 ROIs) with abnormal score

The cumulative percentages of the number of abnormal FA, MD, AD, RD scores in both MTBI groups (and the TC group), are presented in Table 8. Using chi square analyses, there were no significant differences in the percentage of participants with complicated versus uncomplicated MTBIs that had multiple abnormal FA, MD, AD, and RD scores. The largest differences were found for the number of abnormal MD scores when using three, four, and five or more abnormal scores as a criterion. However, these differences were not statistically significant (Fishers Exact Test, range: p = .147 to p = .211

**Table 8:** Base Rates of the Number of Abnormal DTI-ROI Scores by group.

	Fractional Anisotropy			Axial Diffusivity			Radial Diffusivity			Mean Diffusivity		
# Abnormal Scores	Trauma Control	Uncomp MTBI	Comp MTBI	Trauma Control	Uncomp MTBI	Comp MTBI	Trauma Control	Uncomp MTBI	Comp MTBI	Trauma Control	Uncomp MTBI	Comp MTBI
15 or more^1^	-	3.2	-	-	-	-	3.0	3.2	3.2	-	3.2	3.2
14 or more	-	3.2	3.2	-	-	-	3.0	3.2	3.2	-	3.2	3.2
13 or more	-	3.2	3.2	-	-	-	3.0	3.2	3.2	-	3.2	3.2
12 or more	-	3.2	3.2	-	-	-	3.0	3.2	6.5	-	3.2	3.2
11 or more	-	3.2	3.2	-	-	-	3.0	3.2	6.5	-	3.2	6.5
10 or more	-	3.2	3.2	-	3.2	-	3.0	6.5	6.5	-	3.2	6.5
9 or more	3.0	9.7	3.2	-	3.2	-	3.0	6.5	9.7	-	3.2	6.5
8 or more	3.0	9.7	3.2	-	3.2	-	3.0	9.7	9.7	-	3.2	9.7
7 or more	3.0	9.7	3.2	-	3.2	-	6.1	9.7	9.7	-	3.2	9.7
6 or more	3.0	9.7	6.5	-	3.2	-	6.1	9.7	9.7	3.0	6.5	12.9
5 or more	3.0	9.7	12.9	6.1	6.5	6.5	6.1	9.7	16.1	3.0	6.5	22.6
4 or more	6.1	12.9	16.1	9.1	12.9	12.9	6.1	16.1	16.1	6.1	9.7	25.8
3 or more	9.1	12.9	22.6	15.2	16.1	19.4	9.1	19.4	29.0	12.1	12.9	29.0
2 or more	18.2	41.9	35.5	24.2	29.0	32.3	15.2	45.2	45.2	15.2	32.3	32.3
1 or more	42.4	54.8	51.6	39.4	48.4	54.8	36.4	58.1	54.8	27.3	51.6	41.9
0 scores	100.0	100.0	100.0	100.0	100.0	100.0	100.0	100.0	100.0	100.0	100.0	100.0

Note: N = 62. TC = Trauma Controls; Uncomp = Uncomplicated; Comp = Complicated

^1^Table has been limited to 15 or more scores.

### Comparison of MTBI Groups to Trauma Controls

The uncomplicated MTBI group was compared to the trauma control group on the number of abnormal areas of FA, MD, AD, and RD scores. There were significant main effects (using Mann Whitney U tests) for RD (p = .034, d = .30), but not for FA (p = .147), AD (p = .508), or MD (p = .061). However, although not significantly different (likely due to small sample sizes), medium effect sizes were present for FA (d = .40) and MD (d = .39).

The cumulative percentages of the number of abnormal FA, MD, AD, RD scores for the uncomplicated MTBI group were also compared to the trauma control group. Using Chi Square analyses, there were some significant group differences in the percentage of patients that had multiple abnormal scores for FA, RD, and MD, but not AD. For example, 41.9% of the uncomplicated MTBI group had two or more abnormal FA scores compared to 18.2% of the trauma control group (p = .038). Similarly, (1) 45.2% of the uncomplicated MTBI group had two or more abnormal RD scores compared to 15.2% of the trauma control group (p = .009), and (2) 51.6% of the uncomplicated MTBI group had one or more abnormal MD scores compared to 27.3% of the trauma control group (p = .046).

The complicated MTBI group was also compared to the trauma control group on the number of abnormal areas of FA, MD, AD, and RD scores. There were significant main effects (using Mann Whitney U tests) noted again for RD (p = .047, d = .34), but not for FA (p = .232), AD (p = .272), or MD (p = .112). However, medium effect sizes were again noted for FA (d = .35) and MD (d = .59). In addition, the cumulative percentages of the number of abnormal scores for the complicated MTBI group were compared to the trauma control group. Using Chi Square analyses, there were some significant group differences in the percentage of patients that had multiple abnormal scores for RD and MD, but not FA or AD. For example, 45.2% of the complicated MTBI group had two or more abnormal RD scores compared to 15.2% of the trauma control group (p = .009). Similarly, (1) 25.8% of the complicated MTBI group had four or more abnormal MD scores compared to 6.1% of the trauma control group (p = .041; Fishers exact test), and (2) 22.6% of the complicated MTBI group had five or more abnormal MD scores compared to 3.0% of the trauma control group (p = .025; Fishers exact tests).

## Discussion

It is abundantly clear that objective evidence of intracranial trauma helps to guide immediate treatment decisions and is a powerful predictor of morbidity and mortality in *moderate and severe* TBI[75]. Based on this, the intuitive assumption is that the presence of intracranial abnormalities following a MTBI, as evidenced by day-of-injury CT scan or follow up MRI, would also portend worse outcomes. We hypothesized that the presence of intracranial abnormality on CT or MRI would be associated with worse neurocognitive performance, a greater burden of symptoms, and differences in the microarchitecture of white matter. This hypothesis was based on the reasonable assumption that, on average, the complicated group had greater traumatic force passing through their brains, as evidenced by macroscopic intracranial abnormalities and the longer duration of post-traumatic amnesia. Although the complicated group showed microstructural differences on DTI, they did not report more post-concussion symptoms, greater mental health problems, or perform more poorly on cognitive testing at 6–8 weeks following injury.

This is the first study to compare complicated versus uncomplicated MTBI groups directly using DTI, and to our knowledge the only study to use DTI to compare these groups with trauma controls. Recently the TRACK-TBI group published a DTI study[39] comparing *either* complicated or uncomplicated MTBI patients with *normal* controls, and found that only the complicated group showed a measurable DTI change (a direct complicated-uncomplicated comparison was not done).

Using TBSS, both FA and RD showed significant changes between the complicated and uncomplicated groups, most prominently in the CC, but also in the left frontal corona radiata. The results regarding the CC were expected, because it has been shown to be the anatomical structure most sensitive to DTI changes after injury[40]. The reason for this vulnerability may be both anatomical and technical. Anatomically, the long inter-hemispheric course of the CC makes it susceptible to shearing forces and significant distortion, especially in response to rotational forces. Autopsy studies of traumatic axonal injury confirm that the corpus callosum is particularly prone to injury[76]. The CC’s large size, easily recognizable boundaries, generally constant morphology, and highly organized structure also impart a technical accessibility lacking in other areas. This results in a relatively high signal-to-noise ratio that likely renders the TBSS technique more sensitive to changes within the CC than in other, less uniform areas.

Using TBSS, the frontal white matter tracts also showed significant differences between groups, which is also consistent with the literature. After the corpus callosum, the frontal lobe is the most commonly reported area showing DTI changes after MTBI, irrespective of the DTI method of analysis[40,77]. The vulnerability of the frontal lobes to traumatic injury is multifactorial in nature, and theories advanced include proximity to the skull’s bony protuberances, irregularities in the dura near the falx, and the tendency for deceleration injuries, which are a common mechanism of injury in TBI, to cause frontal lobe injury.

A cursory look at the anatomical distribution of group differences in FA (Fig 1) and RD (Fig 2) reveals obvious similarities. This is expected when one considers what these two parameters are measuring, and that they are interdependent rather than independent measures of diffusion. The mathematical derivation for FA incorporates both perpendicular and parallel eigenvectors resulting in a value representing the *fraction* of diffusion that is in the direction of the white matter tract. Lower FA can therefore be the result of (a) less diffusion in the parallel, or *axial* direction, or (b) greater diffusion in the perpendicular, or *radial* direction. We therefore interpret the decrease in FA in the complicated group to be related to the increase in RD, because these two changes anatomically co-localize, and an increase in RD, in isolation, leads to a decrease in FA. The other possibility to consider is that the decrease in FA was related to a decrease in AD. We, however, found no differences in AD between groups, and thus we cannot conclude that alterations in AD were responsible for the FA findings.

This pattern of subacute DTI changes after MTBI is consistent with most other studies[40,41]. A parsimonious physiological interpretation of these findings is that white matter integrity was compromised (decreased FA) mostly as a result of a change in membrane permeability/demyelination (increased RD) without evidence of axonal compromise (no change in AD; however, for an alternate explanation for normal AD in the subacute period, see MacDonald et al, 2011)[78]. That said, it is worth noting that the magnitude of the difference in DTI findings between those with complicated versus uncomplicated injuries was smaller than we anticipated. We thought that the discrete ROI analyses (i.e., those areas identified as different on TBSS) and multivariate ROI analyses would reveal greater differences between the two MTBI groups because we assumed that greater traumatic force would be exerted on the brains of those with visible intracranial abnormalities. Additional research that examines specific subtypes of injuries (and their associated biomechanics) in relation to specific intracranial abnormalities (e.g., type and location) might help better identify those with complicated injuries who are at risk for sustaining more widespread damage to their brains.

### Clinical Outcome: Cognition, Post-Concussion Symptoms, and Mental Health

This study adds to the complexity of the findings in the literature (see Table 1). More than 20 years ago, William’s et al. observed that complicated MTBI subjects closely resemble their *moderate* TBI counterparts in their recovery trajectory[10]. Similarly, Kashluba et al., in a study of 102 patients with complicated MTBI found that, acutely and at one year, the complicated MTBI group was as impaired cognitively and functionally as a group of moderate TBI subjects[79]. Both study samples, however, are much different than ours—the Kashluba subjects were all admitted to an inpatient rehabilitation facility and the William’s sample was all referred to neurosurgery. Therefore, the subjects in those studies represent a minority of MTBI cases that clearly have more serious brain injuries and functional impairment than those in the current study.

As mentioned in the introduction, several studies report worse neurocognitive outcomes in patients with complicated MTBI, while others do not (Table 1). Omnibus grouping of neurocognitive test results may be less informative than partitioning the studies by known moderators, such as timing of testing post injury and patient recruitment location. If we restrict our comparison to studies that tested mainly civilian outpatients after the acute recovery period, then, with rare exception, traditional cognitive testing is insensitive to macrostructural intracranial abnormalities in MTBI (see Table 1), presumably due to spontaneous biological recovery.

A number of studies, such as ours, challenge the assumption that visible intracranial abnormalities in MTBI, *in toto*, are predictive of symptoms. De Guise et al., in a study of 176 MTBI patients, found that those with positive CT scan findings also reported *fewer* post-concussive symptoms, and they showed no difference on neuropsychological evaluations compared to those with negative scans[31]. The authors speculated that perhaps a heightened metacognition and awareness of symptoms, due to the *absence* of an objective lesion, might have led to an *increase* in symptom reporting in the uncomplicated group[31]. Other possiblities include a greater degree of reassurance given to complicated MTBI patients by medical staff in the face of an evident CT abnormality[31]. Iverson et al. studied 47 patients with MTBI and found that although patients in the complicated group took significantly longer to return to work (36 days vs 6 days), they showed a trend toward *lower scores* on scales measuring post-concussion and depression symptoms, and they were not more likely to meet ICD-10 criteria for post-concussion disorder[3].

There is a large literature suggesting that many biopsychosocial factors[60,80,81,82,83,84,85] other than macroscopic or microscopic trauma-related anatomical changes, influence the person’s experience with, and reporting of, post-concusssion and post-concussion-like symptoms. The manifestation of post-concussion symptoms likely represents the cumulative effect of multiple variables, such as genetics, mental health history, current life stress, general medical problems, chronic pain, depression, substance abuse, and iatrogenic influences. How people report their symptoms can also be influenced by personality factors and by the presence of possible future financial gain (e.g., personal injury litigation or disability determinations). In the largest prospective study to date McMahon and colleagues found that patients without abnormalities on day-of-injury CT scan were just as likely to report symptoms three months post injury, and significantly *more* likely to report symptoms at 6 and 12 months post injury[36]. The authors do not speculate as to reasons for the increased symptom reporting in uncomplicated patients, but do note that the differences at six and twelve months disappeared when patients with past histories of psychiatric or neurological problems, drug use, or previous TBI were excluded[36].

One possible contributing factor for the general lack of support for a difference in symptoms in those with complicated versus uncomplicated MTBI is that the taxonomy is simply too broad to be useful. For example, a patient with a small occipital subdural hemorrhage may recover very differently than one with generalized diffuse axonal injury, yet these two are both simply categorized as complicated injuries. Recent studies addressing this issue have dissected the complicated group into various component pathologies, and some have found a correlation with outcome. Examples include worse outcome in the context of diffuse axonal injury[8,34] and parenchymal contusions[8,39].

### Limitations

There are several important limitations to this study. For example, we made no attempt to balance our groups on pre-injury psychiatric dimensions, which may have modified our results, and there are other pertinent factors that we did not consider. A non-exhaustive list includes presence of legal issues, premorbid personality, previous history of brain injury, and psychological coping styles[82]. In addition, there are gender differences in outcome from brain injury; we did not have a large enough sample to examine this. It is also possible that the time of 6–8 weeks for MRI and DTI assessment may not be as informative as in the acute or chronic stages. We await a maturation of the literature to answer this important question. Finally, the different sensitivities of MRI and CT are well known, yet our complicated group was comprised of participants with abnormalities visible with *either or both* methods. Use of both modalities to define what constitutes a traumatic neuroimaging abnormality (ie. a “complicated” MTBI) is fairly common as reflected by the number of studies using both modalities (see Table 1). This does, however, create a more heterogeneous group with regard to severity than only relying on positive CT findings, with the latter expected to be a more severe group given the lesser sensitivity of this imaging modality. Directly comparing our results to studies relying only on CT must, therefore, be done with the caveat that our complicated group as a whole is likely to include a milder injury subset, and thus bias our findings in the direction of a lesser effect than had we used day-of-injury CT only. Further dividing the complicated group into CT- and CT+ subgroups would yield too small a sample size for meaninful comparisons on DTI metrics or neurocognitive and symptom evaluations, and thus was not pursued in the present study (although this could be pursued in future studies). Finally, the symptomatic participants in this study may be different than those who might be symptomatic in the chronic period. In addition, diffusion imaging parameters change at various stages of recovery. The pattern of DTI changes over time may therefore be more likely to relate to clinical outcomes than a static cross-sectional evaluation. A longitudinal analysis incorporating multiple imaging time points would be especially helpful in answering this question.

## Conclusions

In conclusion, this study examined outcome from complicated versus uncomplicated MTBI using symptom ratings (post-concussion and mental health), comprehensive cognitive testing, and DTI at approximately 6–8 weeks following injury. The groups did not differ in their ratings of post-concussion symptoms, depression, or anxiety. Moreover, they did not differ in their performance on a battery of neuropsychological tests. There was a non-significant trend for those in the complicated MTBI group (25.8%) to have more low scores on cognitive testing (i.e., three or more scores below the 10^th^ percentile; Table 6) than the uncomplicated MTBI group (12.9%). If this finding replicates or remains with a stable finding with a larger sample, it could reflect a small embedded subgroup within the complicated MTBI sample that has worse cognitive outcome. Following this group over a longer period of time may also help to address this issue. The two groups also differed in the microstructural integrity of their white matter skeletons, as measured by TBSS, in the corpus callosum and in frontal areas. In the present study, however, the macrostructural and microstructural imaging abnormalities were not associated with worse clinical outcomes at 6–8 weeks following injury. Follow up of these subjects at a later time point will be important for a future study to determine whether or not the uncomplicated and complicated groups show different recovery trajectories beyond the subacute period, on imaging or other clinical outcome measures.
